# Selected renal cells harbor nephrogenic potential

**DOI:** 10.3389/fmed.2022.1062890

**Published:** 2022-12-22

**Authors:** Prakash Narayan, Andrew T. Bruce, Elias A. Rivera, Timothy A. Bertram, Deepak Jain

**Affiliations:** Department of Bioprocess Research and Development, ProKidney LLC, Winston-Salem, NC, United States

**Keywords:** cell, therapy, organoids, kidney, disease, nephrogenic, tubules, glomeruli

## Abstract

Selected renal cells (SRCs), a renal epithelial cell-enriched platform, are being advanced as an autologous cell-based therapy for the treatment of chronic kidney disease. However, the mechanism underlying its renal reparative and restorative effects remains to be fully elucidated. In this study, we coupled knowledgebase data with empirical findings to demonstrate that genes differentially expressed by SRCs form interactomes within tubules and glomeruli and mediate a suite of renal developmental activities including epithelial cell differentiation, renal vasculature development, and glomerular and nephron development. In culture, SRCs form organoids which self-assemble into tubules in the presence of a scaffold. Implanted into the kidneys of subtotally nephrectomized rats, SRCs are associated with comma- and S-shaped body cell formation and glomerular development, and improvement in renal filtration indices and renal microarchitecture. These data suggest that SRCs harbor nephrogenic potential, which may explain, at least in part, their therapeutic activity.

## Introduction

Driven by the diabetes and metabolic syndrome epidemics, chronic kidney disease (CKD) has emerged as a major health and socioeconomic burden worldwide (1–4). Management of CKD remains a challenge for the nephrologist, evidenced by the increasing need for renal replacement therapy. Many patients reach end-stage renal disease (ESRD) having become refractory to regimen that correct disease etiology. Hemodialysis, the mainstay for management of ESRD, carries risks of cardiovascular and cerebrovascular accidents and infections (5), and is a bridge to organ transplantation. Given the relative paucity in the number of donor kidneys available for transplant, there is urgent need for novel therapeutics that target and improve the renal microarchitecture, offering the potential for tissue repair and restoration of function.

Autologous cell therapy employs an individual’s cells, which typically are cultured and expanded outside the body and then introduced into the donor (6). Advantages of such an approach include reduced risks for alloimmune events, bioincompatibility, and disease transmission (6). Autologous selected renal cell (SRC) therapy involves use of biopsy-sourced kidney cells that are expanded in culture, submitted to gradient separation followed by implantation of a select fraction into the donor’s kidney (7–13). Phenotype-labeling studies suggest that SRCs are composed primarily of renal epithelial cells, including podocytes (7–13), a finding consistent with the source of SRCs, viz. the renal cortex. Numerous reports indicate that the tubular epithelium has the capacity to regenerate, repair, and re-epithelialize in response to insult (14–16). Recent evidence suggests that podocytes harbor a distinct mechanism for repopulation as well (17, 18). Consistent with these observations, non-clinical data indicate that administration of SRCs to diseased kidneys stimulates a reparative response and improvement in renal filtration (8–10, 12). Evaluation of glomerular filtration rate in a subset of diabetic kidney disease (DKD) patients at increased risk for ESRD suggests that randomization to SRCs (REACT™) is associated with stabilization of renal function (13).

Transcriptomic analyses indicate overexpression of certain mRNA by SRCs (10). These mRNA, differentially expressed vs. the source biopsy, include cadherin 1/*cdh1*, cubulin/*cubn*, nephrin/*nphs1*, erythropoietin/*epo*, kinase domain region/*kdr* [vascular endothelial growth factor (VEGF) receptor gene], hairy and enhancer of split-1/*hes1*, and platelet endothelial cell adhesion molecule/*pecam1* (10). In the present study, we coupled knowledgebase data with empirical results from *in vitro and in vivo* assays to elucidate a potential mechanism of action underlying reparative and restorative activity of SRCs.

## Results

**FIGURE 1:**
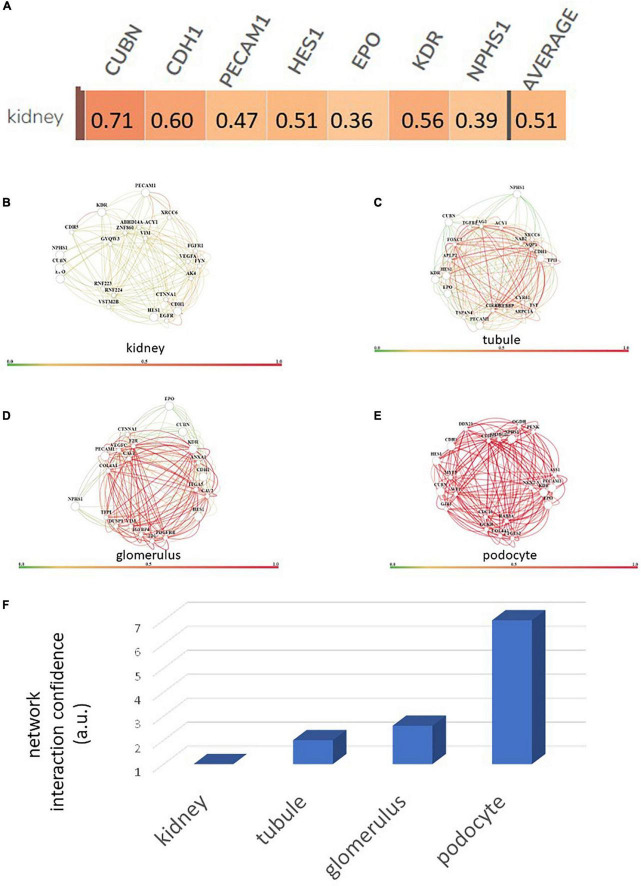
Renal expression of selected renal cell (SRC) genes and their interactomes. Each of the SRC mRNA is expressed by the kidney with an average confidence of 0.51 **(A)**. Interactomes are formed by these mRNA together with other mRNA in the kidney **(B)**, tubules **(C)**, glomeruli **(D)**, and podocytes **(E)**. Interaction strength confidence for these networks is kidney < tubule < glomerus < podocyte **(F)**.

**FIGURE 2:**
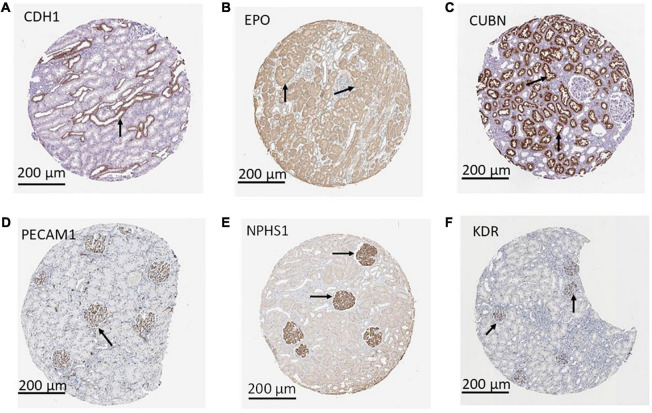
Compartmentalization of selected renal cell (SRC) gene products. Antibody imaging data housed in the human tissue atlas indicate compartmentalization (black arrows) of SRC gene products. Staining for CDH1 antibody is punctate and prominent in the collecting ducts and distal tubules **(A)**. Staining for the EPO **(B)** and CUBN **(C)** antibodies is localized to the tubules whereas PECAM1 **(D)**, NPHS1 **(E)**, and KDR **(F)** antibodies exhibit punctate glomerular staining.

Using a targeted transcriptomic approach, we previously reported that SRCs differentially express *cubn*, *cdh1*, *pecam1*, *hes1*, *epo*, *kdr*, and *nphs1* (10). We queried Humanbase (19) to determine whether these mRNA form interactomes within the kidney. Each of these mRNA is indeed expressed by the kidney (Figure 1A), suggesting that it is not a product of the SRC preparation process. Predictive analytics coupled with empirical data indicate that while these mRNA form interactomes within the renal compartments (Figures 1B–E) strength of their interaction confidence profiles for kidney < tubules < glomeruli < podocytes (Figure 1F). These data suggest that cross-talk between these nodes is likely compartmentalized, occurring primarily within the tubular and glomerular compartments. The Human Protein Atlas (20) houses antibody-imaging based distribution profiles for gene products. Immunohistochemical mapping data indicate that staining for CDH1 antibody (Figure 2A) is punctate and prominent in the collecting ducts and distal tubules. Staining for the EPO (Figure 2B) and CUBN (Figure 2C) antibodies is localized to the tubules, whereas PECAM, NPHS1 and KDR antibodies exhibit punctate glomerular staining (Figures 2D–F, respectively). These gene product distribution profiles, consistent with the SRC interactome profiles, also point to compartmentalization of SRC activity within the tubules and glomeruli.

**FIGURE 3:**
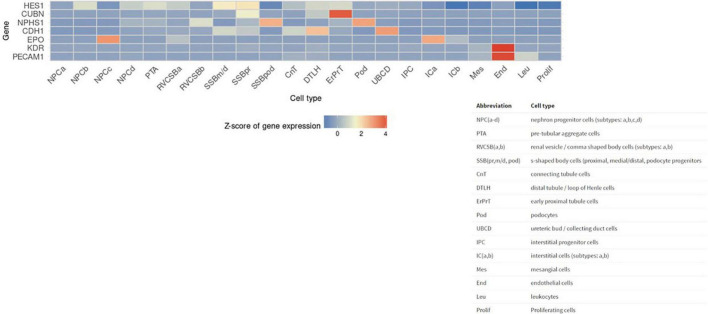
Selected renal cell (SRC) genes are also expressed by renal progenitors. Human Fetal Kidney Atlas heat map showing cells involved in fetal kidney development that also expresses SRC genes.

**FIGURE 4:**
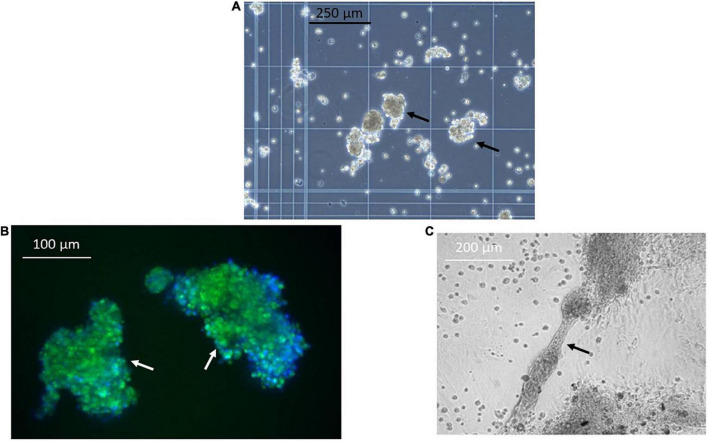
Selected renal cells (SRCs) form organoids and tubules. Representative image obtained using phase contrast microscopy showing formation of organoids (black arrows) **(A)** by SRCs in culture. SRC-derived organoids express the tubular marker chemokine receptor 4 (CXCR4) (white arrows) **(B)**. Representative image showing tubule formation by SRCs in culture (black arrow) **(C)**.

**FIGURE 5:**
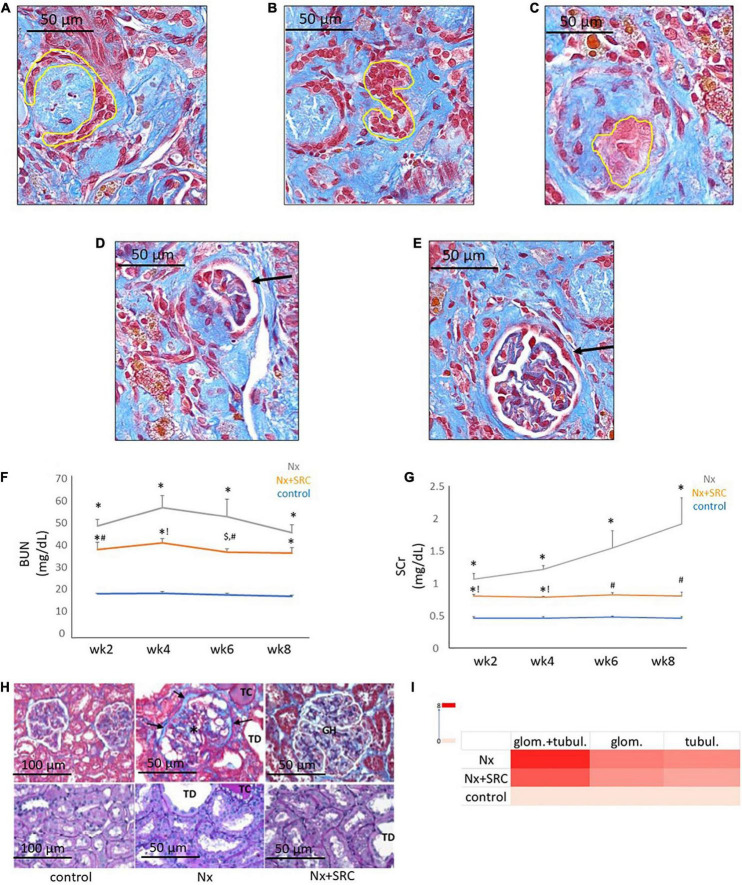
Activity of selected renal cells (SRCs) *in vivo*. Representative day 5 renal sections from rats submitted to Nx and administered SRC exhibiting the different stages of glomerulogenesis (yellow outline or black arrow) can be seen, including commashaped body cell **(A)**, S-shaped body cell **(B)**, the capillary loop phase **(C)**, and the maturing glomerulus **(D,E)**. Nx was associated with an increased BUN **(F)** at weeks 2, 4, 6, and 8 following randomization (*p < 0.01 vs. sham control). Compared to the Nx cohort, treatment with SRCs was associated with reduced BUN levels at weeks 2 (#*p* < 0.05 vs. Nx; *p < 0.01 vs. sham control), 4 (!*p* < 0.01 vs. Nx; *p < 0.01 vs. sham control) and 6 (#*p* < 0.05 vs. Nx; $*p* < 0.05 vs. sham control) following randomization. At 8 weeks following randomization, BUN levels were not significantly different vs. Nx (*p* = 0.057; *p < 0.01 vs. sham control). Nx was associated with increased SCr **(G)** at weeks 2, 4, 6, and 8 following randomization (*p < 0.01 vs. sham control). Compared to the Nx cohort, treatment with SRCs was associated with reduced SCr levels at weeks 2 (#*p* < 0.05 vs. Nx; *p < 0.01 vs. sham control), 4 (!*p* < 0.01 vs. Nx; *p < 0.01 vs. sham control), 6 (#*p* < 0.05 vs. Nx; *p* > 0.05 vs. sham control) and 8 (#*p* < 0.05 vs. Nx; *p* > 0.05 vs. sham control). Representative month 6 renal sections **(H)** from sham control, Nx and Nx + SRC cohorts. The Nx cohort exhibits severe focal segmental glomerulosclerosis, glomerular atrophy, adhesions of sclerotic segment to Bowman’s capsule (arrows), and shrinkage of capillary tufts (*), accompanied by tubular dilatation (“TD”) and tubular casts (“TC”). By contrast, the Nx + SRC treated kidney exhibits reduced glomerular changes, consisting predominantly of compensatory glomerular hypertrophy (“GH”), characterized by enlargement of glomeruli without appreciable injury. In addition, SRC treatment is associated reduced tubular and glomerular injury vs. the Nx cohort **(I)**.

Next, we sought to identify functional mechanisms governing the tissue-reparative activity of SRCs. Seeding the SRC mRNA into Humanbase and gene ontology (GO) biological process (BP) (21) indicates that these nodes participate in a suite of kidney development activities, including epithelial cell differentiation, renal vasculature development, and glomerular and nephron development (Table 1). Not surprisingly, query of the Human Fetal Kidney Atlas (22) indicates that these mRNA are also expressed by cells involved in embryologic kidney development, including nephron and podocyte progenitor cells, s- and comma-shaped body cells, pre-tubular aggregate cells and early proximal tubule cells (Figure 3). These data suggest that SRCs harbor nephrogenic potential, which we next evaluated in proof-of-concept *in vitro* and *in vivo* assays. Placed in culture, SRCs formed organoids that express the tubular phenotypic marker chemokine receptor 4 [CXCR4, (23)] (Figures 4A, B), and when cultured in the presence of hydrogel, these SRC-derived organoids formed tubules (Figure 4C). Interventional effects of SRCs were evaluated in the rat model of sub-total nephrectomy (Nx). Five days following SRC administration into the renal cortex of Nx rats, glomerular development, including comma- and S-shaped body cell formation (Figures 5A, B, respectively) and capillary loop stage (Figure 5C), advancing to maturing stages of glomerular morphogenesis was observed (Figures 5D, E). Treatment with SRCs did not affect mean arterial pressure (MAP; 97 ± 3 mmHg in control; 119.8 ± 8 mmHg in Nx; 106 ± 9 mmHg in Nx + SRC; p > 0.05). Compared to the control cohort, rats submitted to Nx exhibited an increase in blood urea nitrogen (BUN) and serum creatinine (SCr) (Figures 5F, G, respectively). Randomization to SRCs was associated with improved renal filtration evidenced by reduced BUN and SCr levels (Figures 5F, G, respectively). Masson’s trichrome and periodic acid Schiff (PAS)-stained sections were evaluated in each group. Minimal histological changes were observed in kidneys from the control cohort (Figure 5). The remnant kidney in the Nx cohort was characterized by glomerular and tubulointerstitial injury (Figures 5H, I). Glomerular pathology included severe focal segmental glomerulosclerosis, glomerular atrophy, adhesions of sclerotic segment to Bowman’s capsule, and shrinkage of capillary tufts, accompanied by tubular dilatation and tubular casts. Dilated tubules with an accumulation of proteinaceous casts were prominent, as were tubular atrophy and tubulointerstitial fibrosis. By contrast, SRC treatment was associated with reduced glomerular changes, consisting predominantly of compensatory glomerular hypertrophy characterized by enlargement of glomeruli without appreciable injury, reduced tubular dilatation and protein accumulation, and markedly reduced tubulointerstitial fibrosis (Figures 5H, I).

**TABLE 1:** Gene ontology (GO) biological process (BP) analysis of selected renal cell (SRC) genes. Genes expressed by SRCs participate in processes critical to development of the kidney.

Biological processes (BP)
Regulation of kidney development
Epithelial cell differentiation
Kidney epithelium development
Kidney vasculature development
Nephron development
Nephron epithelium development
Glomerulus development
Glomerulus vasculature development
Rental filtration cell differentiation
Glomerulus development
Epithelial cell differentiation

## Discussion

**FIGURE 6:**
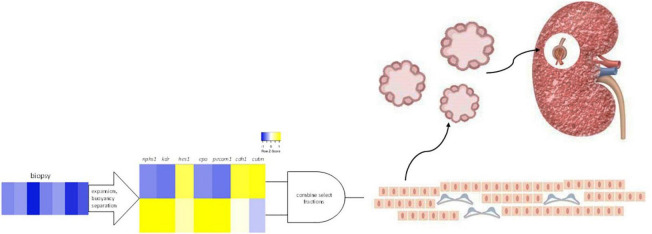
Selected renal cells (SRCs). Rat renal cortical cells are expanded and subjected to buoyancy separation. Two bands differentially expressing *nphs1*, *kdr*, *hes1*, *epo*, *pecam1*, *cdh1*, and *cubn* are selected and combined to produce rat SRCs. Culturing of SRCs results in the formation of organoids and implanted into the diseased kidney, SRCs are associated with nephron development and improvement in renal function and tissue microarchitecture.

SRCs, a renal epithelial cell-enriched platform, are being advanced as autologous cell-based therapy for the treatment of CKD (13). In the present study, we report that the gene products of *cdh1*, *cubn*, *nphs1*, *epo*, *kdr*, *hes1*, and *pecam1*, mRNA differentially expressed by SRCs (10), reside within the tubular and glomerular compartments. Interactomes formed by these nodes also appear localized within the tubules and glomeruli and are involved in a suite of kidney developmental activities. Culturing of SRCs results in formation of organoids, and in the presence of a scaffold, these SRC organoids assemble into tubules. Implanted into the diseased kidney, SRCs are associated with nephron development, and improvement in renal function and tissue microarchitecture. These data suggest that SRCs harbor nephrogenic potential, which may underlie its reparative and restorative effects (Figure 6).

SRCs (REACT™) represent autologous cell-based therapy—a platform that has been deployed to bioengineer skin substitutes, aid wound healing, counteract chronic inflammation, treat burns and pressure ulcers, and improve postoperative healing (6). Derived from a corticomedullary biopsy, SRCs are prepared via a multistage process (U.S. Patent 8,318,484), including enzymatic dissociation of renal tissue followed by buoyancy separation of the cells. Two cellular fractions are selected and combined to form rat SRCs, which comprise primarily renal epithelial cells, including podocytes (7–12). Extensive non-clinical evidence corroborated by emerging clinical data support the notion that orthotopic implantation of SRCs are reparative and restorative (8–10, 12, 13). In the ZSF1 rat model of DKD (12), intervention with SRCs improved survival and mitigated loss of renal structure and function. Emerging Phase II clinical data from a subset of diabetic patients at increased risk for ESRD suggests that randomization to SRCs are associated with with stabilization of renal filtration (18). To delineate the mechanism of action underlying SRC activity in the present study coupled data from search engines, including Humanbase (19), GO BP (21), and tissue atlases (20, 22), with results from *in vitro* and *in vivo* assays. HumanBase and GO BP learn biological associations from extensive data collections while modeling tissue- and BP-specific gene interactions by leveraging experimentally verified tissue expression and gene function. Tissue atlases, repositories of protein expression profiles at single cell and subcellular resolution, marry spatial biology with function. Query of these knowledgebases were associated with three independent findings united by a consistent and repetitive theme. Genes expressed by SRCs viz. *cdh1*, *cubn*, *nphs1*, *epo*, *kdr*, *hes1*, and *pecam1*, appear to form interactomes within the tubules and glomeruli. Second, SRC gene products are localized to these renal compartments. Third, SRC genes participate in a suite of kidney developmental activities. Epithelial cell differentiation, renal vasculature development, and glomerular and nephron development are outputs of these genes or of networks formed by these genes. That SRCs recapitulate events associated with embryologic kidney development is supported by the observation that renal progenitor cells also express these genes.

The hallmark finding of this study is proof-of-concept for the nephrogenic potential of SRCs. In culture, SRCs forms organoids characterized by the expression of a tubular marker CXCR4, a receptor involved in epithelial patterning, tubulogenesis and renal morphogenesis (23). Indeed, inactivation of this receptor in embryonic kidney explants results in impaired ureteric bud branching and mesenchymal tubulogenesis, and renal malformation (23). Consistent with the role of this receptor, SRC organoids, in the presence of a scaffolding material like hydrogel, assemble into tubules. Indeed, tubule formation in the presence of a scaffold involves budding and branching morphogenesis (24). The primary role of the scaffolding material is to act as an extracellular matrix (ECM) and overcome variations in differentiation capacity inside growing organoids, allowing them to survive, grow and proliferate (24). The ECM typically has tiny pores that allow the passage of nutrients and gasses to give the cells the environment they need to thrive. The ECM also provide essential cues to cells rendering them critical for the establishment of physiologically relevant 3D tissue cultures (24). Several important observations emerge from these data. First, SRC derived from the kidneys of obese ZSF1 rats, a model of progressive DKD (12), retains the capacity for organoid formation and tubular assembly. Second, SRC organoids appear committed to renal development, unlike induced pluripotent stem cells that can mature into a non-renal cell (25). These observations are clinically relevant from the perspective of use in a DKD patient population and test article safety (25). Of significance, administration of SRCs was associated with beneficial effects in the rodent Nx model of kidney disease. Randomization to SRCs was associated with improvement in BUN and SCr, clinically reported measures of renal filtration. Accompanying these physiologic findings was a preservation of renal microarchitecture with treatment benefits observed across both the tubulointerstitial and glomerular compartments. Since there were no observed hemodynamic changes associated with SRC therapy, these treatment effects were likely directly related to mitigation of renal injury. Furthermore, interventional benefit was observed in female rats atop the protection afforded by endogenous estrogen (26), a significant finding in terms of the demographics likely to benefit from this therapy. Administration of SRCs into the kidney was accompanied by nephrogenesis characterized by hallmark features including comma- and S-shaped bodies and maturing glomeruli (27, 28). Sourced from the renal cortex, the SRC manufacturing process involves cell expansion, buoyancy separation, and selection of tubular epithelial cells and podocytes (7, 10). Numerous studies have documented the innate capacity of the tubular epithelium to regenerate following insult (14–16). Indeed, renal epithelial regeneration is thought to be mediated by the actions of scattered tubular cells/renal progenitor cells and/or via dedifferentiation of certain tubules (14, 15). Recent studies indicate that the podocyte compartment is dynamic and harbors a mechanism for podocyte replenishment (16, 17). These cell populations with reparative capacity while presumably endogenous to every kidney may lack critical mass in CKD and/or find themselves in an ECM-rich environment not amenable to their activation. The cell expansion and selection process associated with its manufacture makes SRCs a conduit/platform carrying a critical and enriched mass of renal epithelial cells, including podocytes to restitute kidney microarchitecture and function. Indeed, this is borne out by the differential expression of mRNA with nephrogenic potential.

SRCs (REACT™) are being evaluated in a Phase 3 clinical trial in subjects at increased risk (CKD 3b/4) for ESRD. The present study suggests that SRCs are a standalone platform harboring nephrogenic potential, which may form the basis, at least in part, for its reparative and restorative activity.

## Materials and methods

### Gene ontology and mapping

Heat maps for *cdh1*, *cubn*, *epo*, *kdr*, *nphs1*, *pecam1*, and *hes1* were generated using Heatmapper (29) from previously published data (10). Genes were seeded into Humanbase (19) to identify renal tissue expression of these genes, networks within the kidney, and tubular and glomerular compartments. Both Humanbase and GO BP (21) were used to identify kidney-relevant processes. SRC gene products were seeded into The Human Protein Atlas (20) for renal distribution of these proteins. Data on HES1 are not available within this repository. SRC genes were seeded into the Human Fetal Kidney Atlas (22) to identify other cells that also express these genes.

### Preparation of SRCs for cell culture studies

All procedures involving animals were conducted in accordance with National Institutes of Health guidelines and were approved by the Institutional Animal Care and Use Committee (IACUC) of Charles River Labs (MA). Complete methodology for SRC preparation has been previously described (10). Briefly, SRCs were derived from whole kidneys from six to ten weeks old female ZSF1 obese rats (Charles River). Kidneys were enzymatically digested and unfractionated cells and separated on an iodixanol (OptiprepTM, Sigma Aldrich, MO, USA) discontinuous density gradient to manufacture SRC.

### SRC culture

SRCs were cultured as described previously (30). Cells were stained with the renal marker CXCR4 (MAB21651-SP, R&D Systems) and visualized using immunofluorescence microscopy. For 3-dimensional culture in hydrogels, up to 10^6^ SRCs were resuspended in a 50/50 mixture of collagen(I)/collagen(IV) and seeded into individual wells of a six-well cell culture dish. Once the suspension had hardened, the SRC hydrogel was layered with 3 ml renal cell culture medium. Cultures were allowed to develop for up to 2 weeks post-seeding or until evidence of *de novo* formation of 3D structures.

### Preparation of SRCs for *in vivo* studies

The preparation of SRCs has been described extensively (7, 10). Briefly, two-week-old male Lewis rats were sacrificed, and kidneys were harvested at Hilltop Labs (Scottsdale, PA, USA) under its institutional guidelines. Freshly excised tissue was placed into 50 ml conical tubes (10 kidneys per tube) containing 50 ml of cold (4°C) Hypothermasol (Biolife Solutions, Inc. Bothell, WA, USA) and shipped to ProKidney (Winston-Salem, NC, USA) on ice for next-day delivery. Upon receipt, the tubes containing kidneys were cleaned with 70% ethanol and placed in a biological safety cabinet for processing. The initial isolation of a heterogenous primary cell culture population (UNFX) obtained from digested whole rat kidneys was performed using standard protocols (7, 10). Cell fractions were generated by placing a cell suspension generated from the UNFX primary culture onto a four-step iodixanol (OptiPrep^®^) (AxisShield, Norton, MA, USA); density gradient layered as 16, 13, 11, and 7% iodixanol in a 15 ml conical polypropylene tube and centrifuged at 800 × *g* for 20 min at room temperature and washed thrice in sterile phosphate-buffered saline (PBS) prior to use. This process separates cells based on their buoyant density into five distinct bands/fractions (B1–B5). Bands were washed thrice in sterile PBS. SRCs were prepared by combining the B2 and B4 cellular fractions at a ratio of 97% B2:3% B4 as a suspension (5 × 10^6^ cells/100 μl) in sterile PBS. SRCs were stored at (4°C) for >18 h prior to administration.

### Rat model of CKD

The surgical model was undertaken by Charles River Laboratory (Wilmington, PA, USA) after IACUC protocol approval (#2285). Female Lewis rats (8-10 weeks of age) were subjected to a 2-step 5/6 surgical nephrectomy (Nx) (31). Rats were administered buprenorphine (Buprenex, 0.3 mg/ml, intraperitoneally) and then anesthetized via isoflurane inhalant anesthetic by first placing in an isoflurane chamber at 4–5%, then maintaining anesthesia with isoflurane inhalant (3%) via nose-cone throughout the procedure. Each animal was given a second dose of Buprenex after surgery, and a third dose the following day. Anesthesia was confirmed by absence of toe-pinch reflex, and the left dorsolateral area was shaved using a number 5 clipper and cleaned thoroughly with betadine (three times) and ethanol (four times). Sterile transparent adhesive drapes were applied to the surgical area to provide an aseptic environment and enable accurate monitoring of respiration. A ventral midline incision into the abdomen, and the intestine retracted laterally to expose the animal’s left kidney. The kidney was freed from the surrounding tissue. A piece of suture was placed around each pole of the kidney at its one-third position. The sutures were gently ligated around the kidney. A third of the kidney at each pole was excised right beyond the ligatures. The abdominal incision was closed with suture and wound clips. One week after the first step, the animal was anesthetized and prepared as described above. A cranial-caudal skin incision was made on the animal’s right lateral to the spine with its cranial terminus just behind the rib cage. The abdominal cavity was accessed. The right kidney was freed from the surrounding tissue and excised. The incision was closed with suture and wound clips. Animals were returned to their cages and allowed to recover. A control group comprised animals with both kidneys intact. At the end of the in-life component of the study, animals were placed in a CO_2_ chamber. Death was confirmed by the absence of deep pain reflex and the ocular test.

### Administration of SRCs

Six control and twelve subtotally Nx animals were entered into the study. Two weeks after excision of the contralateral kidney, the remnant left kidney was accessed through a longitudinal incision in the left dorsolateral area. The kidney was isolated and partially extracted from the peritoneal space using sterile gauze and blunt surgical forceps. SRC (5 × 10^6^ cells in 100 μl PBS) loaded into a single sterile 1 mL syringe (BD, Franklin Lakes, NJ, USA), fitted with a 23 G needle was delivered slowly through the needle into the kidney parenchyma, approximating the corticomedullary junction area. Upon delivery of SRCs (n = five animals), the injection site was compressed with sterile forceps as the needle was withdrawn to slow bleeding and reduce loss of injected material. The kidney was returned to the peritoneal space and 1 mL of warm sterile saline was added for hydration. The fascia was closed using 4.0 Vicryl sutures and the skin was closed with wound clips (Ethicon Inc., Somerville, NJ, USA for both items). Oxygen was administered post-surgery, and animals were monitored until alert and conscious.

### MAP

Systolic and diastolic blood pressure was measured using a CODA non-invasive tail-cuff monitor (Kent Scientific, Torrington, CN, USA) and MAP calculated as the mean of the systolic and diastolic blood pressure values.

### Renal function and histopathology

Blood urea nitrogen (mg/dl) and SCr (mg/dl) were measured starting on weeks 2, 4, 6, and 8 post-treatment. Serum samples were submitted to a core laboratory (Antech, Morrisville, NC, USA) for the clinical chemistry measurements. At sacrifice (5 days or 6 month after randomization to SRCs), the kidney was removed and bisected coronally. Paraffin-embedded 5 μm tissue sections were stained with Masson’s trichrome and periodic acid Schiff (PAS). Evaluation of remnant kidney parenchyma was performed by light microscopy. Tubulointerstitial and glomerular injury indexes were scored with standard semi-quantitative grading scales of 0–4 (worsening) as described previously (10).

### Data analysis

Data were expressed as mean ± standard error or mean and analyzed by one-way analysis of variance followed by Tukey’s *post-hoc* test. A *p* < 0.05 was considered statistically significant.

## Data availability statement

The original contributions presented in this study are included in the article/supplementary material, further inquiries can be directed to the corresponding author.

## Ethics statement

This animal study was reviewed and approved by Charles River Labs.

## Author contributions

PN, AB, and ER performed the studies and analyzed the data. TB and DJ oversaw the project. PN wrote the manuscript. AB, ER, TB, and DJ revised the manuscript. All authors contributed to the article and approved the submitted version.
